# Cancer mortality in a cohort of asbestos textile workers

**DOI:** 10.1038/sj.bjc.6602240

**Published:** 2005-02-08

**Authors:** E Pira, C Pelucchi, L Buffoni, A Palmas, M Turbiglio, E Negri, P G Piolatto, C La Vecchia

**Affiliations:** 1 Dipartimento di Traumatologia, Ortopedia e Medicina del Lavoro, Università degli Studi di Torino, via Zuretti 29, 10126 Torino, Italy; 2 Istituto di Ricerche Farmacologiche ‘Mario Negri’, via Eritrea 62, 20157 Milano, Italy; 3 Istituto di Statistica Medica e Biometria, Università degli Studi di Milano, via Venezian 1, 20133 Milano, Italy

**Keywords:** asbestos, cancer mortality, cohort study, lung cancer, mesothelioma

## Abstract

A cohort of 889 men and 1077 women employed for at least 1 month between 1946 and 1984 by a former Italian leading asbestos (mainly textile) company, characterised by extremely heavy exposures often for short durations, was followed up to 1996, for a total of 53 024 person-years of observation. Employment data were obtained from factory personnel records, while vital status and causes of death were ascertained through municipality registers and local health units. We observed 222 cancer deaths compared with 116.4 expected (standardized mortality ratio, SMR=191). The highest ratios were found for pleural (SMR=4105), peritoneal (SMR=1817) and lung (SMR=282) cancers. We observed direct relationships with duration of employment for lung and peritoneal cancer, and with time since first employment for lung cancer and mesothelioma. Pleural cancer risk was independent from duration (SMR=3428 for employment <1 year, 7659 for 1–4 years, 2979 for 5–9 years and 2130 for ⩾10 years). Corresponding SMRs for lung cancer were 139, 251, 233 and 531. Nonsignificantly increased ratios were found for ovarian (SMR=261), laryngeal (SMR=238) and oro-pharyngeal (SMR=226) cancers. This study confirms and further quantifies the central role of latency in pleural mesothelioma and of cumulative exposure in lung cancer.

Cohort studies found increased incidence and mortality from mesothelioma among (chiefly male) asbestos workers employed in mining (Piolatto *et al*, 1990; McDonald *et al*, 1993), but mainly textile and manufacture (Newhouse and Berry, 1979; Berry *et al*, 2000; Yano *et al*, 2001), insulation (Selikoff *et al*, 1979) and asbestos-cement factories (Tulchinsky *et al*, 1999; Ulvestad *et al*, 2002). Mesothelioma risk varied widely among different studies, according to types of asbestos involved (chrysotile, amosite or crocidolite) (Hodgson and Darnton, 2000) and levels of exposure (Doll and Peto, 1985). Since other factors such as duration and years since first and last asbestos exposure (Bianchi *et al*, 1997; Hauptmann *et al*, 2002) also determine mesothelioma risk, it is important to analyse all these factors to disentangle their separate effects. This could also help to understand the recent levelling off of mesothelioma trends in some European countries (Peto *et al*, 1999; Hemminki and Li, 2003; Montanaro *et al*, 2003; Pelucchi *et al*, 2004).

Asbestos is a major cause of lung cancer too, acting multiplicatively with tobacco smoking (Saracci, 1977; Vainio and Boffetta, 1994). A meta-analysis of 69 cohort studies found a standardized mortality ratio (SMR) of 163 for lung cancer, with a substantial heterogeneity (Goodman *et al*, 1999) largely attributable to the different cumulative exposure to asbestos in various cohorts, this being the major determinant of lung cancer risk (Boffetta, 1998). Chrysotile fibres are considered less carcinogenic than amphiboles and it has been estimated that cohorts exposed to amosite and crocidolite experience an excess of lung cancer in the exposure specific risk of around 5% per ff ml^−1^ year, while the corresponding risk for chrysotile alone is between 0.1 and 0.5% (Hodgson and Darnton, 2000).

To further investigate these issues, we have analysed data from a cohort including about 900 men and more than 1000 women from an Italian company. Sited in Grugliasco, near Turin, the company produced various asbestos-containing, particularly textile, goods.

## MATERIALS AND METHODS

Subjects included in the cohort were 1973 men and women who had worked in a major asbestos textile manufacture for at least 1 month in the period from 1946 to 1984, when production was dismissed. Seven subjects (0.4%) lacked information on date of birth or date of death and were thus excluded from the analysis, leaving 1966 subjects (889 males and 1077 females) who were followed up to 31 December 1996 or until reaching 80 years of age, if this occurred earlier. Overall, 1333 (67.8%) subjects survived the follow-up period, 545 (27.7%) died and 88 (4.5%) emigrated or were lost to follow-up for other reasons. Subjects lost to follow-up were included in the analysis, considering the last information available as end point. A total of 53 024 person-years of observation (22 332 man-years and 30 692 woman-years) were covered.

Employment data were obtained from personnel records at the factory, while vital status and causes of death were ascertained through registers from municipal offices and local health units. Information was available on date and place of birth, migration(s) and death, on cause of death and on periods and duties of employment(s). Cases of mesothelioma were further checked matching them up to regional mesothelioma registers.

We computed the expected numbers of deaths from all causes and from selected cancer sites using national death rates for each 5-year calendar period and age group. National death rates for each cause and quinquennium of age were available from 1955 and were taken from the Central Institute of Statistics (ISTAT, 1955–1996). We therefore applied the 1955–1959 death rates also to periods 1946–1949 and 1950–1954. SMR were computed for the overall study population as well as according to duration, time since first and last employment and age at first employment, separately for men and women (Breslow and Day, 1987).

Only the date of death, and not the date of diagnosis, was available to us. As retirement or change of job may in some cases have been due to the diseases themselves, deaths that occurred within 3 years of stopping exposure were considered together with those during exposure.

Two general models were considered for the mesothelioma death rates in workers exposed to asbestos (Breslow *et al*, 1983). These were, first, the multiplicative (or relative risk) model:

*O*_*(k)*_*=E*_*(k)*_ e^*β′Z(k)*^

where *O*_*(k)*_ is the observed number of mesothelioma deaths in the *k*th category, *E*_*(k)*_ is the expected number of deaths in the *k*th category based on national mortality rates, *Z*_*(k)*_ is a vector of covariates (such as age at first exposure, duration, etc) whose influence on the event is being examined, and *β*′ is a vector of unknown parameters, to be estimated. In this model, the effect of each factor acts multiplicatively on the expected rate in the general population.

The second general model considered was the additive (or excess risk) model:

*O*_*(k)*_*=E*_*(k)*_*+PY*_*(k)*_e^*β′Z(k)*^

with variables defined as above, and *PY*_*(k)*_ defined as the total number of person-years in the category *K*. In this model, each factor is assumed to act multiplicatively on the excess risk, and the resulting product then adds to the expected number in the general population. To fit the two equations, we used the GLIM package (Baker and Nelder, 1978).

Various types of asbestos were used in the factory, including crocidolite. To estimate levels of asbestos exposure, environmental sampling was carried out (Scansetti *et al*, 1985) as follows: static samples were taken between 1973 and 1978/9, using mercury clepsydra vacuum pump (‘Zurlo’) with mixed-cellulose ester filters (MCE), Micropore, 20 mm diameter (diameter filter efficacy 6 mm) and 0.8 *μ*m porosity at a capturing speed of 1.2 m s^−1^. Sampling time was from 15 to 20–25 min for a volume of air from 1 to 2 l. Personal samples were taken from 1978 using the ‘Du Pont alpha 1 model’ and ‘Zambelli Ego model’ constant flow instrumentation. A 40-mm long cylinder-shaped spacer was used together with MCE, Micropore, 25 mm in diameter and porosity of 0.8 *μ*m. Flow rate was 2 l min^−1^ for 30–50 min.

The MCE filters were cleared, in both cases, using acetone vapours with a few drops of Triacetin (Glycerol Triacetate) to spread fibres evenly on the filter. Phase contrast optical microscopy (× 500) was used to count the fibres. Only respirable fibres were counted using a Walton-Beckett graticulate. Respirable fibres were defined as diameter ⩽3 *μ*m; length ⩾5 *μ*m; aspect-ratio (length–diameter relationship) at least 3 : 1, and UICC standards were used for crysothile, crocidolite, amosite and anthophyllite asbestos.

## RESULTS

Table 1 gives observed number of deaths and the SMRs for several cancer sites, separately for men and women. Overall, we observed 222 cancer deaths compared with 116.4 expected (SMR=190.7). The highest ratios were observed for pleural (SMR=4105.5) and peritoneal cancers (SMR=1817.2). Excess ratios were found also for lung, ovarian, laryngeal and oro-pharyngeal cancers. The SMR for all neoplasms were 204.6 for women and 184.5 for men. For pleural, peritoneal and lung cancer the SMR was higher in women, while no female deaths were observed for oral, pharyngeal and laryngeal cancers. There were 38 deaths attributable to asbestosis. The SMR for pleural cancer is probably overestimated, since national mortality rates were used to compute expected deaths. In the mid-1970s the province of Turin had, compared with the whole of Italy, SMR for pleural cancer of 187 for males and 191 for females. Correcting for these ratios, the SMR for pleural cancer would be about 2200. On the other hand, for most cancer sites of interest, Turin rates did not differ importantly from Italian ones. In fact, the SMR for all neoplasms for the province of Turin as compared to Italy was 109 for males and 114 for females (Cislaghi *et al*, 1986).

Cancers of the respiratory and digestive tracts and of the ovary are considered, for men and women combined, in Table 2 in relation to duration of employment. For total cancer mortality and for mortality overall, the SMR increased with duration of employment. For lung cancer, the SMR rose from 139.1 for exposures <1 year to 530.9 for exposures ⩾10 years. A direct relationship was observed also for peritoneal (the highest SMR was 4298.0 for employment ⩾10 years), but not for pleural and gastrointestinal cancers. The SMR for pleural cancer was 3427.7 for <1 year of exposure, 7659.3 for 1 to <5 years, 2978.6 for 5 to <10 years and 2129.9 for ⩾10 years.

Corresponding figures with relation to time since first employment are given in Table 3. The SMR for all causes of death increased with time since first employment. For all neoplasms combined, the SMR was 79.6 for <15 years since first exposure, 217.3 for 15 to <25, 245.8 for 25 to <35 and 217.2 for ⩾35 years. Between 15 and 25 years since first employment, the SMR were 1599.4 for peritoneal cancer and 2910.2 for pleural and peritoneal mesothelioma combined; corresponding values increased to 2062.9 and 3593.0 for latency of 25 to <35 years, and to 5360.6 and 4480.7 for latency ⩾35 years. There were 12 deaths from pleural cancer 25 years or more after first employment, while 0.3 were expected.

Time since last exposure is considered in Table 4. No significant excess in overall mortality (SMR=103.3) nor in cancer mortality (SMR=107.3) was reported during employment. However, elevated SMR were observed in almost all subgroups of pleural and peritoneal cancers, peaking at 4736.8 after 15 to <25 years since last employment for mesotheliomas.

Table 5 reports SMR for selected cancer sites in subgroups of age at first employment. No evident pattern emerged, the SMR were higher for overall mortality (184.2), peritoneal (4183.8) and pleural cancers (6562.3), and mesothelioma (5110.7) when first employment occurred before 25 years of age, while the SMR were higher for all cancers (216.8) and for lung cancer (408.3) in the subgroup of first employment occurring between 25 and 35 years of age.

Table 6 shows the parameter estimates and their standard errors obtained when selected covariates of interest were simultaneously fitted to mesothelioma deaths, under multiplicative and additive models. Also, Table 6 reports the estimates for each variable in relation to one of the categories of the variable chosen as reference. A test for the significance of each parameter can be made by comparing the ratio of the parameter to its standard error to a standard normal deviate. Both relative and excess risks were closely related with time since first and last employment, the additive model showing greater estimates. Age at first exposure was inversely related with mesothelioma risk under the multiplicative model, while excess risk showed an elevated risk in subjects aged 35 or older when starting exposure. Duration of employment showed no clear pattern of risk, though the risk estimates were consistently above unity under both models.

**Figure 1:**
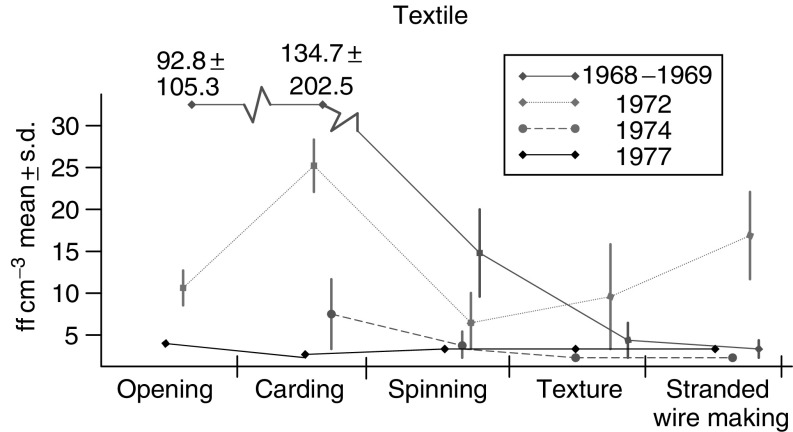
Level of asbestos exposure in departments in the factory.

Figure 1 reports levels of asbestos dusts in various departments of the factory. In carding, levels as high as 100 ff cm^−3^ were found in the late 60s and as high as 25 ff cm^−3^ in the early 70s.

## DISCUSSION

This cohort study of asbestos textile workers found an extremely high SMR of pleural and peritoneal mesothelioma. Increased SMRs were also observed for lung, ovarian, laryngeal and oral and pharyngeal cancers, as well as for all cancer mortality that was approximately twice than expected. Overall, observed deaths were 60% more than expected, due to excess mortality from cancers but also from other respiratory tract diseases. The large number of deaths from asbestosis testify the magnitude of exposures.

Relative risks of mesothelioma following asbestos exposure have varied widely in previous studies. Two recent cohort studies in Norway and Israel found standardized incidence ratios above 5000 (Tulchinsky *et al*, 1999; Ulvestad *et al*, 2002). The risk of developing mesothelioma has been estimated as proportional to a third or fourth power of time since first occupational exposure to asbestos (Peto *et al*, 1982). In this study, the SMR of mesothelioma was 2780, and when we considered selected time-related covariates together in a model, latency was the main determinant of mesothelioma risk. There was no significant trend in risk with duration of employment, and also exposures of a few months increased pleural cancer risk. In fact, six out of 23 individuals deceased for pleural cancer had an employment period lower than 1 year, and three of those worked for less than 4 months at the company. We found high relative and absolute risks for a long time since last employment. However, time since first and last employment were highly correlated – due to short duration of exposures reported for several members of this cohort – and it was not possible to consider these variables together to disentangle their separate effects.

These findings are compatible with models of carcinogenesis involving an early stage effect for asbestos exposure (Peto *et al*, 1982). On the other side, we observed increasing absolute risks with age at first exposure. This may be due to unreported earlier exposures and/or to residual confounding. In any case the excess risk was an order of magnitude lower than that reported for latency.

With reference to other neoplasms, asbestos workers of the textile industry have been reported to experience the highest lung cancer risks, probably because of the stronger carcinogenic effect of long and thin fibres used (Mossman and Gee, 1989; Boffetta, 1998). We found a substantial increase of lung cancer risk (SMR=282), with a direct trend with duration of employment, besides latency. The SMR of lung cancer increased up to 25 years after exposure had ceased, and then declined. As a result, the excess deaths from mesothelioma surpassed those from lung cancer ⩾25 years after last employment (9.7 *vs* 7) and ⩾35 years after first employment (8.8 *vs* 6.5).

In this study, oro-pharyngeal and laryngeal cancers presented similar results: seven male and no female deaths, and 2.3-fold elevated SMR, comparable to other Italian asbestos-exposed cohorts (Piolatto *et al*, 1990; Puntoni *et al*, 2001). Since these findings may simply reflect bias or confounding (Griffiths and Malony, 2003), we computed expected deaths and SMR for male laryngeal cancer also applying Piedmont regional mortality rates, which were available from 1970 but the SMR decreased only slightly, to 213.5. However, an elevated consumption of alcohol and cigarettes in the cohort studied as compared to the general population could not be excluded, since no information was available on these aspects for most subjects. Inferences from relative risks of other illnesses are difficult, since smoking and asbestos exposures generally affect the same diseases, that is, those of the respiratory tract. Considering alcohol intake, we used mortality from liver cirrhosis as an indicator of consumption. We observed 19 male deaths from liver cirrhosis, while 14.2 were expected (SMR=134). Further, a potential effect of tobacco and alcohol is supported by the observation that all oro-pharyngeal, laryngeal and liver cancer deaths were among males, and that cigarette smoking has been uncommon among Italian women until recent years (Gallus *et al*, 2002).

The excess risk of ovarian cancer (five observed deaths, SMR=261) can be related to the exposure of ovarian epithelium which, like the peritoneum, is exposed to the carcinogenic effect of asbestos (Parazzini *et al*, 1991).

Intestinal cancers also had increased SMR, but potential mis-diagnoses of these cancer sites instead of nearby peritoneal mesothelioma remain possible (Doll and Peto, 1987). Considering the small number of deaths and the weakness of the association, 2–3 mis-diagnosed deaths would be enough to explain the elevated SMR. It should be noted, however, that data from literature do not fully agree on the association between the above cancers, mainly laryngeal, gastrointestinal and ovarian cancer and asbestos exposure (Doll and Peto, 1985; Zheng *et al*, 1992; Consensus Report, 1997; Rafferty *et al*, 2001; De la Provote *et al*, 2002; Griffiths and Malony, 2003).

We found no excess risk of most urogenital tract cancers, particularly of the kidney (SMR=98) that has been reported as a potential asbestos-related cancer (Smith *et al*, 1989; Consensus Report, 1997).

The latency period between first asbestos exposure and occurrence of mesothelioma is seldom less than 10 years (Doll and Peto, 1985), while we found that two male subjects had died from pleural cancer only 6 years after first employment. However, both of them reported previous job-related exposures to asbestos. No other pleural cancer and one out of 14 peritoneal cancer cases reported previous occupational exposures. This was of concern, because the analysis on time-related covariates could have been affected. However, duration of employment of the three subjects who died from mesothelioma was less than 1, 3 and 6 years, respectively. One started working at the company at 29 years and two after 35 years of age. Therefore, it is possible that mesothelioma risks for a late age at first exposure could be somewhat overestimated.

Among the strengths of the study are the high number of women included, the originality of the cohort, which included subjects exposed to very high doses of asbestos fibres, and the long follow-up that allowed to adequately evaluate risk of mesothelioma, that is, a cancer with a long latency period between first asbestos exposure and cancer death.

This study, in conclusion, provides additional quantitative information on the relative risks of pleural and peritoneal mesothelioma (including ovarian cancer) and lung cancer in a cohort of textile workers. The central role of latency and of cumulative exposure in determining, respectively, the risk of mesothelioma and of lung cancer was also assessed. Of specific interest is the over 30-fold excess risk of pleural cancer even in workers exposed for very short time (<1 year). In fact, exposure in this factory almost approached experimental conditions.

## Figures and Tables

**Table 1:** Observed mortality and standardized mortality ratios (SMR), with 95% confidence intervals (CI), of selected cancers

	**Males**		**Females**		**Total**		
**Cause of death (ICD-IX)**	**Obs**	**SMR**	**Obs**	**SMR**	**Obs**	**SMR**	**95% CI**
Oral and pharyngeal (140–149)	7	253.9^a^	0	0	7	225.6	90–465
Stomach (151)	11	115.9	4	134.3	15	120.3	67–198
Colorectal (152–154∣159.0)	10	139.2	6	156.1	16	145.1	83–235
Liver (155)	6	225.7	0	0	6	177.9	65–388
Peritoneum (158)	5	1090.3^a^	9	2886.5^a^	14	1817.2^a^	992–3053
Larynx (161)	7	245.8	0	0	7	238.0	95–490
Lung (162)	62	252.4^a^	14	595.6^a^	76	282.4^a^	222–354
Pleura (163)	12	2851.5^a^	11	7891.2^a^	23	4105.5^a^	2603–6158
Breast (174)	0	0	7	82.2	7	81.5	33–168
Ovary (183)	—	—	5	261.4	5	261.4	85–609
Prostate (185)	2	60.4	—	—	2	60.4	7–218
Bladder (188)	3	93.5	0	0	3	85.3	18–249
Kidney (189)	0	0	2	424.1	2	98.0	12–354
Brain (191–192)	4	150.0	1	61.6	5	116.6	38–272
							
Pleura+peritoneum	17	1933.1^a^	20	4432.6^a^	37	2780.7^a^	1958–3833
							
All cancers (140–239)	148	184.5^a^	74	204.6^a^	222	190.7^a^	166–218
							
Asbestosis (501)	27	—	11	—	38	—	—
							
All causes	377	154.0^a^	168	178.2^a^	545	160.7^a^	147–175
							
Total no. of subjects	889		1077		1966		
Person-years	22 332		30 692		53 024		

a 95% confidence interval does not include 100.

**Table 2:** Observed mortality and standardized mortality ratios (SMR) of selected cancers, according to duration of employment (years)

	**<1**		**1 to <5**		**5 to <10**		**⩾10**	
**Cause of death**	**Obs**	**SMR**	**Obs**	**SMR**	**Obs**	**SMR**	**Obs**	**SMR**
Oral and pharyngeal cancer	4	388.7^a^	2	252.3	0	0	1	132.6
Stomach cancer	5	142.4	2	62.6	6	263.6	2	57.3
Colorectal cancer	7	223.4	1	35.2	3	145.5	5	167.2
Peritoneal cancer	1	468.4	1	494.8	3	2063.1^a^	9	4298.0^a^
Laryngeal cancer	1	105.4	3	397.9	2	390.3	1	137.7
Lung cancer	12	139.1	17	250.8^a^	11	232.9^a^	36	530.9^a^
Pleural cancer	6	3427.7^a^	11	7659.3^a^	3	2978.6^a^	3	2129.9^a^
Ovarian cancer	2	440.8	0	0	0	0	3	573.7^a^
								
Pleural+peritoneal cancers	7	1801.8^a^	12	3471.2^a^	6	2437.8^a^	12	3426.1^a^
								
All cancers	51	149.0^a^	50	165.7^a^	41	191.4^a^	80	261.7^a^
All causes	136	138.6^a^	131	148.6^a^	97	156.7^a^	181	199.1^a^
								
Total no. of subjects	671		530		328		437	
Person-years	18 870		16 194		9234		8726	

a 85% confidence interval does not include 100.

**Table 3:** Observed mortality and standardized mortality ratios (SMR) of selected cancers, according to time since first employment (years)

	**<15**		**15 to <25**		**25 to <35**		**⩾35**	
**Cause of death**	**Obs**	**SMR**	**Obs**	**SMR**	**Obs**	**SMR**	**Obs**	**SMR**
Oral and pharyngeal cancer	3	336.4	4	363.3	0	0	0	0
Stomach cancer	5	136.5	6	147.8	3	100.3	1	56.8
Colorectal cancer	2	85.5	2	54.6	7	223.7	5	264.2
Peritoneal cancer	0	0	4	1599.4^a^	4	2062.9^a^	6	5360.6^a^
Laryngeal cancer	1	106.3	1	98.1	5	732.3^a^	0	0
Lung cancer	6	90.1	28	295.1^a^	32	439.3^a^	10	287.4^a^
Pleural cancer	2	1872.5^a^	9	4578.4^a^	9	5359.8^a^	3	3373.3^a^
Ovarian cancer	0	0	2	332.1	1	163.8	2	537.2
								
Pleural+peritoneal cancers	2	622.5	13	2910.2^a^	13	3593.0^a^	9	4480.7^a^
								
All cancers	23	79.6	85	217.3^a^	77	245.8^a^	37	217.2^a^
All causes	96	100.5	193	176.2^a^	154	184.5^a^	102	201.6^a^
								
Total no. of subjects	170		384		933		479	
Person-years	25 263		16 065		8672		3023	

a 85% confidence interval does not include 100.

**Table 4:** Observed mortality and standardized mortality ratios (SMR) of selected cancers, according to time since last exposure (years)

	**During exposure/<3**		**3 to <15**		**15 to <25**		**25 to <35**		**⩾35**	
**Cause of death**	**Obs**	**SMR**	**Obs**	**SMR**	**Obs**	**SMR**	**Obs**	**SMR**	**Obs**	**SMR**
Oral and pharyngeal cancer	1	186.4	2	178.9	4	472.0^a^	0	0	0	0
Stomach cancer	2	86.8	6	128.6	4	120.9	2	119.9	1	189.7
Colorectal cancer	0	0	5	134.4	6	183.2	3	151.7	2	291.0
Peritoneal cancer	1	736.9	2	728.1	10	4791.6^a^	0	0	1	2785.5
Laryngeal cancer	0	0	3	271.3	2	266.6	2	498.5	0	0
Lung cancer	7	183.2	30	309.3^a^	26	351.9^a^	11	242.3^a^	2	136.5
Pleural cancer	1	1733.1	5	2718.9^a^	8	4670.2^a^	8	7252.9^a^	1	2702.7
Ovarian cancer	1	415.5	2	366.0	1	172.3	0	0	1	747.9
										
Pleural+peritoneal cancers	2	1034.1^a^	7	1526.4^a^	18	4736.8^a^	8	3547.7^a^	2	2743.5^a^
										
All cancers	19	107.3	82	202.4^a^	75	230.3^a^	34	176.2^a^	12	190.4
All causes	60	103.3	206	169.8^a^	168	179.3^a^	85	171.6^a^	26	158.1^a^
										
Total no. of subjects	139		395		510		703		219	
Person-years	14 493		20 254		12 027		5182		1068	

a 85% confidence interval does not include 100.

**Table 5:** Observed mortality and standardized mortality ratios (SMR) of selected cancers, according to age at first exposure (years)

	**<25**		**25 to <35**		**⩾35**	
**Cause of death**	**Obs**	**SMR**	**Obs**	**SMR**	**Obs**	**SMR**
Oral and pharyngeal cancer	0	0	2	257.1	5	262.0
Stomach cancer	2	131.4	1	37.6	12	144.8
Colorectal cancer	3	163.2	2	75.3	11	168.3
Peritoneal cancer	6	4183.8^a^	6	3072.3^a^	2	463.3
Laryngeal cancer	1	383.8	1	157.2	5	244.5
Lung cancer	8	279.5^a^	25	408.3^a^	43	239.9^a^
Pleural cancer	6	6562.3^a^	5	3512.6^a^	12	3675.9^a^
Ovarian cancer	2	275.6	2	337.2	1	168.5
						
Pleural+peritoneal cancers	12	5110.7^a^	11	3258.3^a^	14	1846.5^a^
						
All cancers	40	203.7^a^	61	216.8^a^	121	176.3^a^
All causes	96	184.2^a^	136	175.1^a^	313	149.5^a^
						
Total no. of subjects	839		514		613	
Person-years	24 269		14 695		14 060	

a 85% confidence interval does not include 100.

**Table 6:** Parameter estimates and their standard errors (s.e.) obtained by fitting selected variables together to risk of mesothelioma (pleural and peritoneal)

				**Multiplicative model**			**Additive model**	
**Covariate**	**Observed deaths**	**Person-years**	**Parameter**	**s.e.**	**RR**	**Parameter**	**s.e.**	**Abs ER**
*Sex* a								
Male	17	22 332	—	—	1.00	—	—	1.00
Female	20	30 692	0.52	0.35	1.68	−0.10	0.25	0.90
*Age at first exposure* a(years)								
<25	12	24 269	—	—	1.00	—	—	1.00
25–34	11	14 695	−0.30	0.42	0.74	0.52	0.29	1.68
⩾35	14	14 060	−0.55	0.45	0.58	1.08	0.30	2.94
								
*Duration of employment* a(years)								
<1	7	18 870	—	—	1.00	—	—	1.00
1 to <5	12	16 194	0.66	0.48	1.93	0.75	0.33	2.12
⩾5	18	17 960	0.37	0.45	1.45	0.57	0.31	1.77
								
*Time since first employment* a(years)								
<15	2	25 263	—	—	1.00	—	—	1.00
15 to <25	13	16 065	1.50	0.75	4.46	2.67	0.61	14.51
⩾25	22	11 696	1.56	0.75	4.77	3.66	0.60	39.06
								
*Time since last employment*^b^ *(years)*								
<3	2	14 493	—	—	1.00	—	—	1.00
3 to <15	7	20 254	0.44	0.80	1.56	1.26	0.59	3.52
⩾15	28	18 277	1.46	0.75	4.31	3.24	0.54	25.56

a Estimates from a model including sex, age at first exposure, duration and time since first employment.

b Estimates from a model including sex, age at first exposure, duration and time since last employment. Relative (RR) and absolute excess risks (ER) are given in relation to an arbitrarily selected reference category.
